# Bayesian models and meta analysis for multiple tissue gene expression data following corticosteroid administration

**DOI:** 10.1186/1471-2105-9-354

**Published:** 2008-08-28

**Authors:** Yulan Liang, Arpad Kelemen

**Affiliations:** 1 Department of Organizational Systems and Adult Health, University of Maryland, 655 W. Lombard Street, Baltimore, MD 21201-1579, USA; 2 Department of Neurology, Buffalo Neuroimaging Analysis Center, The Jacobs Neurological Institute, University at Buffalo, The State University of New York, 100 High Street, Buffalo, NY 14203, USA

## Abstract

**Background:**

This paper addresses key biological problems and statistical issues in the analysis of large gene expression data sets that describe systemic temporal response cascades to therapeutic doses in multiple tissues such as liver, skeletal muscle, and kidney from the same animals. Affymetrix time course gene expression data U34A are obtained from three different tissues including kidney, liver and muscle. Our goal is not only to find the concordance of gene in different tissues, identify the common differentially expressed genes over time and also examine the reproducibility of the findings by integrating the results through meta analysis from multiple tissues in order to gain a significant increase in the power of detecting differentially expressed genes over time and to find the differential differences of three tissues responding to the drug.

**Results and conclusion:**

Bayesian categorical model for estimating the proportion of the 'call' are used for pre-screening genes. Hierarchical Bayesian Mixture Model is further developed for the identifications of differentially expressed genes across time and dynamic clusters. Deviance information criterion is applied to determine the number of components for model comparisons and selections. Bayesian mixture model produces the gene-specific posterior probability of differential/non-differential expression and the 95% credible interval, which is the basis for our further Bayesian meta-inference. Meta-analysis is performed in order to identify commonly expressed genes from multiple tissues that may serve as ideal targets for novel treatment strategies and to integrate the results across separate studies. We have found the common expressed genes in the three tissues. However, the up/down/no regulations of these common genes are different at different time points. Moreover, the most differentially expressed genes were found in the liver, then in kidney, and then in muscle.

## Background

Despite rapid advancements in statistical methods for gene expression microarray analysis, much more work is needed for multiple source heterogeneous genomic data, such as multiple organisms/tissues, multiple platforms, multiple species and even more from transcriptome, genome, to proteome in order to develop valid and dependable methods that are mainly applicable to microarray data. The congruency of these different data sources needs a unified framework for combining the multiple sources and testing associations between them, thus obtaining a robust and integrated view. In the meantime, we may find a surprising discrepancy present elsewhere between gene expressions given multiple source of genomic data sets.

Meta-analysis is a set of statistical procedures designed to integrate experimental and correlational results across independent studies that address a related set of research questions [1-4]. Developing meta-analysis methods for complex biological systems in microarray experiment is important. It can help the global interpretation of results from multiple sources and fully utilize the available data source. Therefore, it appears to be a promising tool that may serve to identify ideal targets for novel treatment strategies, for the resolution of uncertainty, fuzziness, and heterogeneity typically present in genomic data. Moreover, this approach may improve the significance, robustness and efficiency of the statistical inference by incorporating all the available information.

So far a few studies have attempted to integrate the gene expression data sets from different sources in order to yield a model for disease dynamics such as development and behavior. Ghosh et al. discussed the issues of combining the results across various studies using meta-analysis including different experimental platforms [5]. Rhodes et al. applied large scale meta-analysis for cancer microarray data to identify common transcriptional profiles of Neoplastic transformation and progression and illustrated the merits of data sharing [6]. Pan et al. proposed a joint model of multiple types of data that can be employed to use all the data simultaneously to draw inference or make predictions [7]. Conlon et al. proposed the probability integration model for gene expression data and has showed that the model was able to identify more true discovered genes and fewer true omitted genes than combining expression measures [8]. The true integration-driven discovery rate (tIDR) was used to find the common gene sets.

In our earlier studies we have provided detailed reviews of statistical methodologies for time-course gene expression analysis [9-13]. Mixture models have recently become widely used statistical tools in the analysis of heterogeneous data and have been developed to model complex distributions of "target" values of gene expressions, without any dependence on input values for the differential expressions [14-19]. Some of these work has extended two component mixture models to multiple components and utilized EM algorithms and Akaike Information Criterion (AIC) or Bayesian Information Criterion (BIC) as measures for the most preferable number of components [15]. In this paper, we propose a hierarchical mixture model in the fully Bayesian setting for tackling complex biological systems with multiple tissues genomic data sets and conducting meta-analyses to find commonly expressed genes responding to the drug treatment across the tissues.

Corticosteroids are a class of compounds that exhibit the most potent immunosuppressive and anti-inflammatory activities. These drugs are widely used in a variety of acute and chronic disease states, such as asthma, leukemia, and organ transplantation. Although their therapeutic effects result from regulation of immune system genes, many adverse events occur due to unwanted influence of the drug on other genes, primarily those genes involved in metabolic processes [20]. The corticosteroid compounds produce both beneficial, as well as harmful effects, through binding to the same type of glucocorticoid receptor. This binding activity results in a cascade of signal transduction pathways to ultimately produce an eventual drug response and clinical outcome.

Because drug activity requires a sequential series of events in order to elicit its effects, different genes may exhibit different expression profiles over time following the administration of a drug dose. The particular genes that are either up-regulated or down-regulated, in combination with specific time-course patterns and interactions with other genes, may be predictive of the ultimate outcome(s) that result from drug therapy. Therefore, it is important to improve our understanding of the time-dependent changes in gene expression and their interactions caused by corticosteroid therapy in order to potentially discover the precise genes that may be the most important in producing favorable therapeutic outcomes versus those that may instigate negative, unwanted effects. Moreover, all systemic phenomena such as blood pressure involve multiple genes in multiple tissues and pathologies such as diabetes and hypertension are complex phenomena involving altered expression of multiple genes in several tissues.

### Multiple-tissues affymetrix data sets, preprocessing and normalization

Our multiple-tissues/organs time courses affymetrix data sets [20] are from Affymetrix GeneChips^® ^Rat Genome (R_U34A). This is a pre-clinical study performed on experimental rats. There were forty-eight animals that received a single IV bolus 50 mg/kg dose of the anti-inflammatory drug, methylprednisolone (MPL) [21]. Liver, muscle, and kidney tissues were collected from each animal and processed to assay the gene expression. Three rats were consequently sacrificed at each of the following sampling 16 time-points: 0.25, 0.5, 0.75, 1, 2, 4, 5, 5.5, 6, 7, 8, 12, 18, 30, 48, and 72 hours. Triplicate measurements were obtained at each of these time points. Four rats were not administered any drug and were sacrificed at time t = 0, control group. Therefore, totally 52 chips were used in the study for each tissue, each chip has 8799 genes.

To limit potential source of non-biological variation such as those introduced from experimental procedures and to extract real biological variation regarding potentially important changes in gene expression due to MPL dosing, the following procedures will be employed in data quality control and data analysis. To determine expression measures of probe set from probe signals with lowest data variance and bias, we performed one of the most popular probe set algorithms of MAS5  for background subtraction, signal intensity normalization between arrays, and non-specific hybridization correction. By Affymetrix software (MAS 5.0), each probe set in our data assigns an "average difference" value corresponding to the expression level of the particular gene it represents. The calculated average difference is then used as the measure of expression levels and data normalization throughout the data analysis [22]. Gene expressions were converted to a ratio via dividing the gene expression at time *t*_*i *_by the gene expression level at time t_0_, where *i *represents the specific post-dose time-point and *t*_*0 *_represents baseline at time = 0 hours (i.e. the control group that did not receive drug). These ratios were subsequently natural-logarithmically transformed to produce normally distributed gene expression levels at each sampling time-point.

Total RNA was separately extracted from the liver samples from each animal and purified. The isolated RNA was then used to create biotinylated cRNA. Some of these oligonucleotide sequences were from different parts of the same gene (i.e. 5' vs. middle vs. 3' ends of the transcript), but for the most part, each probe identified a unique gene sequence. According to the Affymetrix Microarray Suite 4.0, an initial step was performed to classify signal values as Present (P), Marginal (M), or Absent (A) based on the intensity of the signal [21]. A classification of 'P' means that the signal is reliably detected at a sufficient level to confirm that the gene expression measurement is definitely above background 'noise'. An 'M' represents a signal that may or may not be a result of true gene expression. The 'A' classification signifies an expression level that is near detection limits and cannot be distinguished from background 'noise'. This classification feature was utilized in our Bayesian categorical model for data preprocessing that were implemented prior to hierarchical Bayesian mixture model.

## Results and discussion

### Bayesian categorical model for estimating the proportion of the 'call'

Bayesian categorical model is developed to estimate the proportion of the 'call' information of P, A and M and Bayesian statistical analysis is conducted to filter genes according to the 'call' data by estimating the parameters under multinomial distribution assumption. Genes that have less 'call' of P than expected or more 'call' of A than expected are excluded. We know that the 'call' has three categories. With 3 categories, suppose the counts (n_1_, n_2_, n_3_) have a multinomial distribution with *n *= ∑*n*_*i *_and parameters *π *= (*π*_1_, *π*_2_, *π*_3_)' [23]. Let {p_i _= n_i_/n} be the sample proportion. The likelihood is proportional to $\prod_{i=1}^{3}\pi_{i}^{n_{i}}$. The conjugate density is the Dirichlet, expressed in terms of Gamma functions as

$$\begin{array}{cc} g(\pi )=\frac{\Gamma \left(\sum_{i}\alpha_{i}\right)}{\Pi_{i}\Gamma \left(\alpha_{i}\right)}\Pi_{i}p_{i}^{\alpha_{i}-1} & for\;0<\pi_{i}<1\;all\;i,\sum_{i}\pi_{i}=1. \end{array}$$

where i = 1: Absent; i = 2: Marginal; i = 3: Present

Computations of marginal posterior distributions and their moments could be estimated by simulating samples from them [24]. Markov Chain Monte Carlo (MCMC) method, the stochastic simulation algorithm we choose here, is one of the algorithms to obtain the estimations via sampling and re-sampling procedures and approximating techniques.

### Hierarchical Bayesian Mixture model and meta-analysis

Hierarchical Bayesian mixture model is developed to model the complex distributions of gene expressions [14-17]. The mixture components that are incorporated in the model may be interpretable as representing underlying "latent classes" or dynamic clusters [25-28]. The advantages of Bayesian Finite Mixture Models is that it provides the insights about behavioral patterns as a source of heterogeneity of the various dynamics and can reduce the high dimensionality and make clear the major components of the underlying structure of the data. In this paper, hierarchical Bayesian mixture model is developed to filter out the significantly non-differentially expressed genes across time and find differentially expressed genes across tissues. We model the true density of the time course gene expressions conditional on the observed data as a mixture of densities with more than three components are allowed:

$$\phi \left(x_{it}\right)≅\sum_{j=1}^{C}\theta_{j}f_{j}\left(x_{it}|\alpha_{j}\right)$$

where *x*_*it *_is expression value for the i'th gene at time t, i = 1,...,I, t = 1,...,T. *ϕ*(.) denote the mixture density given the gene expression data. *θ*_*j *_(j = 1,..., C) are component proportions with nonnegative quantities that sum to 1. C is the number of clusters to be determined based on model selection criteria DIC discussed next. *f*_*j *_(*x*_*it *_| *α*_*j*_) is each component density of the mixture.

We denote the parameters in latent cluster j as

$$\begin{array}{c} \begin{array}{cc} \alpha_{j}=\{\mu_{j},\sigma_{i}^{2}\} & j=1,2,...,C, \end{array}\\ \begin{array}{cc} f_{j}(x_{it}|\mu_{j},\sigma_{j}^{2})\sim Normal\left(\mu_{j},\sigma_{j}^{2}\right), & j=1,...,C, \end{array} \end{array}$$

for which the conjugate prior for *μ*_*j *_can be written as

$$\begin{array}{cc} \mu_{j}\sim Normal\left(\gamma_{j0},\sigma_{j0}^{2}/\kappa_{j0}\right), & j=1,...,C. \end{array}$$

The parameters of the prior on *μ*_*j *_are chosen from various values (e.g. 0.001, 0.01, 0.1) to give broad distributions, for instance,

$$\begin{array}{l} \gamma_{j0}\sim Normal(0,0.0001)\\ \sigma_{j0}^{2}\sim Inverse\;Gamma(0.1,0.1) \end{array}$$

Here *γ*_j0 _is an initial guess on the mean in cluster j with *κ*_j0 _a prior sample size reflecting strength of belief in the guess about mean [24].

The precision parameter in cluster j is given by

$$\tau_{j}=1/\sigma_{j}^{2}\sim Inverse\;Gamma(\nu_{j}t_{j}/2,\nu_{j}/2),$$

where *v*_*j *_is the guess of the prior degrees of freedom, typically *v*_*j *_= 2 or lower and *t*_*j *_= 1/$s_{j}^{2}$ is the prior guess at the precision in group j, which will be tested from various values (e.g. 0.001, 0.01, 0.1). The best value will be chosen based on convergence of MCMC.

For the cluster of the genes that are non-differentially expressed across time, we define the mean of this component as 0 (*μ*_1 _= 0). The C clusters in (1) include both non- differentially expressed gene clusters and the clusters with genes with both positive and negative gene expression patterns over time, which are declared differentially expressed clusters. These differentially expressed clusters can be further clustered based on their dynamic changes/patterns over all time points (either up or down regulated across different time points). For C categories components/clusters, the corresponding dynamic patterns can be represented with underlying latent variables. Each underlying latent variable *Z*_*it *_for gene i at time point t is discrete, taking value j = 1,..., C with probability *θ*_j_; *Z*_*it *_~ *Categorical*(*θ*_*j*_). *θ*_j _can follow either parametric prior such as a beta distribution or a Dirichlet distribution or non-parametric Dirichlet Process Prior [24]. In our analysis the component proportions *θ*_j _are assumed to follow Dirichlet prior *θ*_*j *_~ *Dirichlet*(*prior*_*j*_).

Various initial values for prior and hyper-prior distributions for the mean and variances in the mixture model are tested in order to obtain model convergence, such as *μ*_*i *_~ *Normal*(0, 0.0001), $\sigma_{i}^{2}$ ~ *Inverse Gamma *0.1, 0.1). For example., when assigning uninformative distributions to these parameters, i.e. $\sigma_{i}^{2}$ ~ *Inverse Gamma*(0.001, 0.001), the models do not converge. They converged with $\sigma_{i}^{2}$ ~ *Inverse Gamma*(0.1, 0.1).

The computations of marginal posterior distributions and their moments of all the parameters were conducted by MCMC algorithm with Gibbs sampling [29]. Our WinBUGS code is included with this paper [see Additional file 1]. Over-relaxation methods were used to aid the convergence and reduce the chance of local maxima. Semi-heuristically tried various settings of the prior distributions and various initial values for sensitivity analysis, such as the shape and scale parameters were tried with low values 0.001, 0.01, 0.1 and so on. After 1000 times burn in of the MCMC algorithm, the estimates usually converge well, and then we conduct another 1000 iterations to find the estimated quantities of interests.

Spiegelhalter et al. [30] proposed Deviance Information Criterion (DIC) for model selections, which are used in our study for determining the best number of clusters and most appropriate priors. DIC is a new measure for model complexity and goodness of fit under the Bayesian setting. DIC is more appropriate when comparing complex hierarchical models in the Bayesian setting, where the number of parameters is not clearly defined. One advantage is its inclusion of a prior distribution, which induces a dependency between parameters that is likely to reduce the effective dimensionality. DIC is summarized by the posterior expectation of the deviance and complexity (effective number of parameters) as the expected deviance minus deviance at the posterior expectation of the parameters, both calculated from MCMC output.

Our Bayesian mixture model produces the gene-specific posterior probability of differential expression and the 95% credible interval (which covers 95% of the posterior probability distribution), which is more informative than directly conducting hypothesis tests. Hypothesis tests require setting up the null/alternative hypothesis for each tested value of the gene expression, one at a time for the chosen model, which is less efficient than providing confidence interval/credible interval. Most existing works [15-17,19] including some recent works [31,32] in Bayesian mixture models for differential gene expression have focused on doing so, which is one of the major differences between our approaches and these works. Moreover our model provides us the estimates for further Bayesian meta-inference from three independent analyses for three given tissue data sets. Our goal is to gain an increase in the power of detecting dynamically differentially expressed genes and also improve the reproducibility of the findings by integrating the results from different tissues in order to find common targets for evaluating the treatment.

One important feature of the above Hierarchical Bayesian mixture model is that it is appropriate for meta-analysis due to its ability to account for dependence among the genes and pool the means or slopes (together with estimated standard error) of dose-response curves into each cluster/component and thus can summarize the concordance (intersection) among the tissues through the estimated posterior distributions as credible intervals. Therefore, our finite mixture model is used to calculate credible interval for the differentially expressed genes for each tissue. The gene expression measures among tissues are different from one another. However, we have normalized and standardized the measurements to allow comparison between tissues.

Furthermore, our meta-analysis of multiple tissue data here is not simply based on combining gene expression measures across three separate studies, but is based on combining summaries, such as the estimated posterior distributions as credible intervals from our hierarchical mixture models. Conlon et al. showed that the probability integration model identified more true discovered genes and fewer true omitted genes than combining expression measures [8]. The resulting null standard deviations illustrate the precision of the resulting estimates. By combining the resulting estimates based on Bayesian mixture approaches we aim at studying the tissue effects and to find the differential differences of three tissues responding to the drug.

Therefore, hierarchical Bayesian mixture model is conducted for each tissue separately first. This indicates random samples are from three separate distributions not a common population distribution, which is more accurate for approximating and estimating the parameters of the tissues. Then, instead of combining expression measures by including a separate parameter to model the inter-tissue variability for meta-analysis, we compared the resulting estimates of 95% credible intervals from our hierarchical Bayesian model, similar to Conlon et al. [8] with the probability integration model.

### Results from three tissue data

**Figure 1:**
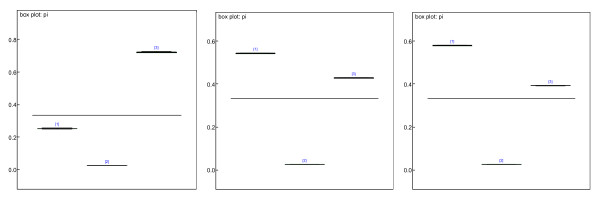
Box-plot of the estimated P_i_s. All the P_i_s are estimated by small variance, which shows the appropriateness of the estimates under assumptions from kidney (left), muscle (middle), liver (right) data.

Bayesian categorical model provided earlier was applied to estimate the proportion of the 'call' for pre-screening genes. After 1000 times burn in via MCMC algorithm, the estimated proportion for each 'call' category p_i_s fluctuated around a value and the variations were stable and tiny, which showed convergence. The densities of the parameters are approximately normally distributed, which show the appropriateness of the prior assumptions of the model. We then conducted another 1000 iterations to estimate proportions (P_i_s, i = 1, 2, 3) of the 'call' of three categories, Present (P), Marginal (M), or Absent (A). Table 1 summarizes the estimates of the P_i_s. Fig. 1 (left) is box-plot of the estimated P_i_s for kidney data. All the P_i_s are estimated with very small standard deviations, which show the appropriateness of the model and the estimates under assumptions. Thus, we conducted filtering step according to this result. All the genes having "call" of A greater than 0.2526*n (n is the number of chips, in our study n = 52) were excluded from our study, as well as the genes having "call" of P less than 0.7224*n (n = 52). 2430 genes from kidney data satisfied the above criteria and were left in our study.

**Table 1:** The estimates of the P_i_s according to the 'call' of P, M and A

	Proportion	Standard Deviation	95% Credible interval
Absent	0.2526	0.001609	(0.2495, 0.2558)
Marginal	0.0250	5.663E-4	(0.02387, 0.02612)
Present	0.7224	0.001692	(0.7192, 0.7257)

**Figure 2:**
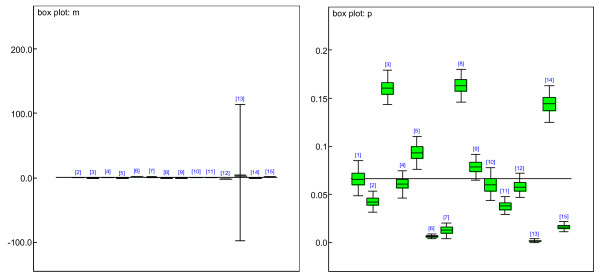
Boxplots of the estimates of means and standard deviations for the mixture of normal distributions (left) and the estimates of proportions of the mixtures (right).

Hierarchical Bayesian Mixture (HBM) model was further applied for the identifications of differentially expressed time related genes and dynamic clusters. We allowed the number of components/clusters in HBM to vary from 5 to 35. Based on DIC and convergence of MCMC, the best model had 15 components. This number of clusters shows the most convergence in all the models with smallest DIC value. We still conducted 1000 iterations (burn ins) via MCMC algorithm, and the estimates converged well. We then processed another 1000 iterations to obtain the estimates of the parameters of HBM. Fig. 2 displays boxplots of the estimates of means and standard deviations for HBM (left). Almost all of them have very small variations, which indicate the appropriateness of the assumptions and estimates. Only one of the mixture groups has means with large variance, because this group includes the outliers, which makes the estimation variance large. The right subfigure sketches the estimated proportions for each mixture component. The variances are small, too. According to the 95% credible intervals, the total percentage of up-regulation is 5.98% among 2430 genes; and the total percentage of down-regulation is 7.85%.

Similarly to kidney data, we applied Bayesian categorical model for estimating the proportion of the 'call' for pre-screening genes for both Muscle (see Table 2 and Fig. 1, middle) and liver tissue data sets (Table 3 and Fig. 1, right). For muscle data, 3849 genes were filtered out using the 'call' information of P, A and M, which were further used by HBM. According to the 95% credible intervals, the total proportion of up-regulation was 1.36% among 3849 genes. For liver data, 3614 genes were filtered out using the 'call' information of P, A and M. The total proportion of up regulation is 17.50% among 3614 genes and 10.40% of the genes were down-regulated in the liver data.

**Table 2:** The estimates of the P_i_s according to the 'call' of P, M and A

	Proportion	Standard Deviation	95% Credible interval
Absent	0.5438	7.379E-4	(0.5423, 0.5452)
Marginal	0.02652	2.382E-4	(0.02607, 0.02701)
Present	0.4297	7.331E-4	(0.4283, 0.4312)

**Table 3:** The estimates of the P_i_s according to the 'call' of P, M and A

	Proportion	Standard Deviation	95% Credible interval
Absent	0.5804	7.621E-4	0.5789, 0.5819
Marginal	0.02622	2.468E-4	0.02575, 0.02673
Present	0.3934	7.54E-4	0.3919, 0.3949

### Pair-wise comparisons between two tissues

Table 4 shows the comparisons of gene expression between kidney data and muscle data that are up/down regulated genes. Similarly, Tables 5 and 6 list the comparisons of gene expressions between kidney data and liver data; and between muscle data and liver data. We reported the differentially expressed genes based on two significant time points: 2 and 6 hours from all three tissue data sets for demonstration to show the up/down regulation patterns of given time points. Moreover, these two selected time points have shown that they are the important/peak time points that were pertinent to drug response. Although selecting the peak/changing time points is not a major focus of this paper, there are many related publications in protein expression studies to achieve such goals. We further cluster gene expressions into up-regulation groups, down-regulation groups and no-regulation groups according to the mean expression level and the credible interval of posterior distributions of the gene expressions obtained for each mixture cluster in order to conduct meta-analysis.

**Table 4:** Comparisons of gene expression between kidney data and muscle data

Kidney	Muscle			Total
	1 outlier	1 up	2 up	
1 down	3	1	1	5
1 outlier	0	0	0	0
1 up	5	6	1	12
1 up 1 down	0	0	0	0
2 up	0	0	0	0
2 down	0	1	0	1

Total	8	8	2	18

CMH test	General association *χ*^2 ^= 2.585, df = 4, P = 0.629			

**Table 5:** Comparisons of gene expressions between kidney data and liver data

Kidney	Liver						Total
	1 down	1 outlier	1 up	1 up 1 down	2 up	2 down	
1 down	25	1	44	6	2	3	81
1 outlier	1	0	1	0	0	0	2
1 up	13	0	39	3	4	4	63
1 up 1 down	0	0	5	0	1	1	7
2 up	0	0	1	0	1	0	2
2 down	2	0	2	0	0	0	4

Total	41	1	92	9	8	8	159

CMH test	General association *χ*^2 ^= 20.912, df = 25, P = 0.698						

**Table 6:** Comparisons of gene expressions between muscle data and liver data

Muscle	Liver						Total
	1 down	1 outlier	1 up	1 up 1 down	2 up	2 down	
1 outlier	2	0	9	0	2	0	13
1 up	4	0	22	0	0	0	26
2 up	1	0	3	1	0	0	5

Total	7	0	34	1	2	0	44

CMH test	General association *χ*^2 ^= 12.814, df = 6, P = 0.0461						

To further examine the congruency and discrepancy between (1) kidney and muscle data; (2) kidney and liver data; (3) muscle and liver data regarding the up/down/no regulation at given time points Cochran-Mantel-Haenszel test was applied, which are reported in Tables 4, 5, 6[33]. The null hypothesis is that there are no associations regarding up/down regulation for two compared tissues for the commonly regulated genes. The calculated p-values for the association between kidney and muscle is P = 0.629 with *χ*^2 ^= 2.585, which indicate no evidence against the null hypothesis of no association. Similar results were found for the kidney and liver (*χ*^2 ^= 20.912, P = 0.698). However, for muscle and liver the *χ*^2 ^= 12.814, df = 6, P = 0.0461, which indicate marginal evidence of association. The number of common expressed genes is relatively small. These findings can be further reevaluated by releasing the stringent family wise error rate to false discovery rate. Results show that the up/down/no regulations of these common genes for compared tissues at different time points are not associated and they can be significantly different.

### Bayesian meta-analysis

**Figure 3:**
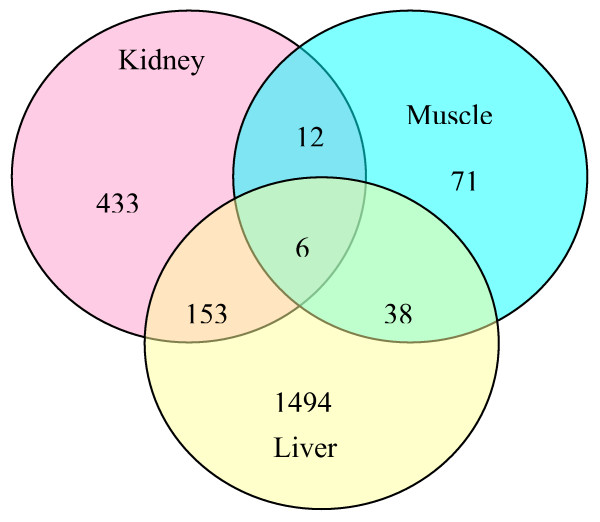
Common gene expressions among three tissues.

**Figure 4:**
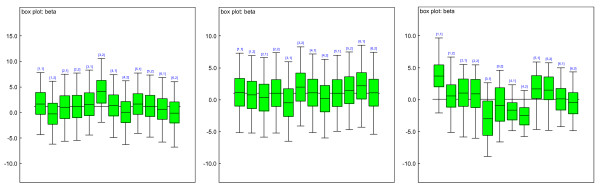
Meta-analysis plots for the gene expressions for the common expressed genes: gene name AF053312_s_at: 2 hrs and 6 hrs; D12769_g_at: 2 hrs and 6 hrs; L19998_at: 2 hrs and 6 hrs; L32591mRNA_g_at*: 2 hrs and 6 hrs; L33869_at: 2 hrs and 6 hrs; U05989_at: 2 hrs and 6 hrs. Each plot, from left to right: kidney, muscle and liver data.

As we have discussed earlier we varied the number of mixtures from 5–35 components for three tissue data sets. Results show that 11–15 provided the most precise and reliable (small CI) results and also the smallest DIC. For further comparisons and meta-analysis, we used 15 as the number of components for final models for all three tissue sets. The gene expressions among tissues were different from one another, which violates the criteria of meta-analysis using pooled data. We analyzed the data tissue by tissue using our proposed HBM and then compared the credible intervals of each gene expression at 2 hrs and 6 hrs among the 6 common genes (see Table 7). Fig. 3 provides the common gene expressions among three tissues. This figure is biologically plausible since muscle is considered to be the most stable tissue among the three and therefore there should be fewer active differentially expressed genes, while the kidney and liver are more active tissues and therefore are more likely to be up/down regulated. All the six common differentially expressed genes among the three tissues were verified by the experiment of Almon, et al. [20]. Fig. 4 shows meta-analysis plots for the gene expressions for the six commonly expressed genes in kidney, muscle and liver and the credible intervals of six gene expressions at 2 hrs and 6 hrs. Each plot, from left to right: gene number 638, 2 hr and 6 hr; 1188, 2 hr and 6 hr; 2150, 2 hr and 6 hr; 2268, 2 hr and 6 hr; 2272, 2 hr and 6 hr; 3517, 2 hr and 6 hr. From those figures, gene number 2150 is up-regulated during the time point of 6 hrs with confidence interval higher than 0 in kidney. Gene number 638, 2150, 2268 and 2272 have significant expression at some time point in liver. We don't find any significant gene expression in muscle. The relatively large confidence intervals are due to the small size of biological replications. We also find that the three tissues have the same tendency of some gene expressions although some of them show significance while others don't (Table 7).

**Table 7:** Clusters and expressions for the genes that expressed among three tissues

Gene number		638	1188	2150	2268	2272	3517
Gene name		AF053312_s_at	D12769_g_at	L19998_at	L32591mRNA_g_at	L33869_at	U05989_at

kidney							
2 hr	Group	7.02	13.86	6.01	15.00	13.02	10.96
	Mean ± std	1.20 ± 1.13	0.08 ± 0.31	2.22 ± 0.43	1.40 ± 0.38	4.11 ± 1.5E8	0.82 ± 0.32
	Expression	No	No	Up	Up	Outlier	Up
6 hr	Group	10.93	14.98	11.87	12.91	14.82	13.48
	Mean ± std	0.82 ± 0.32	1.40 ± 0.38	-1.22 ± 1.14	4.11 ± 1.5E8	1.40 ± 0.38	4.11 ± 1.5E8
	Expression	Up	Up	No	Outlier	Up	Outlier

muscle							
2 hr	Group	13.48	5.33	5.30	12.43	11.42	12.78
	Mean ± std	1.33 ± 0.53	0.24 ± 0.25	0.24 ± 0.25	2.51 ± 0.86	2.51 ± 0.86	9.65 ± 3.2E11
	Expression	Up	No	No	Up	Up	Outlier
6 hr	Group	9.53	10.40	12.26	4.79	13.71	10.31
	Mean ± std	-0.49 ± 0.39	-0.51 ± 1E13	2.51 ± 0.86	0.24 ± 0.25	1.33 ± 0.53	-0.49 ± 0.39
	Expression	No	Outlier	Up	No	Up	No

liver							
2 hr	Group	14.95	4.96	8.48	10.00	4.03	4.99
	Mean ± std	3.66 ± 0.92	2.33 ± 0.51	-0.99 ± 0.64	-5.53 ± 3.11	1.25 ± 0.47	2.33 ± 0.51
	Expression	Up	Up	No	No	Up	Up
6 hr	Group	7.87	5.28	7.90	4.10	3.43	9.23
	Mean ± std	0.68 ± 0.33	2.33 ± 0.51	0.68 ± 0.33	1.25 ± 0.47	-0.60 ± 0.29	-0.99 ± 0.64
	Expression	Up	Up	Up	Up	Down	No

Mean ± std is the base 2 logarithm of the ratio between 2 hrs and baseline, 6 hrs and baseline. If credible interval does not include 0 and is greater than 0, then we consider the genes in this group as up regulated ones; if the interval is below 0, then we consider the genes in this group as down regulated ones; otherwise, they are not regulated.

To further examine the biological functions and interpretations of the discovered commonly expressed genes among three tissues from Fig. 3, we used NCBI web literature search engine. The genes are related to the following possible biological functions: Immune related cluster including interleukin 6 receptor and the interferon gamma receptor gene; Signaling cluster which is dominated by kinases and phosphatases; Transcription cluster related to a major influence of corticosteroids to the transcription; Vascular cluster which contains genes such as the angiotensin converting enzyme; Plasma Membrane cluster which mediates its interaction with the external environment; Protein and Amino Acid Metabolism cluster; Small molecule metabolism and others.

### Simulation and sensitivity analysis

To further determine the validity of the results, we conducted simulation study to ensure that the findings based on our method are valid. We generated "time course gene expression data with 17 unequally spaced time points" with data format similar to our real kidney data for a total of n = 1500 genes. The simulated data was generated from the same normal distribution for error with variances that varied from 0.0001 to 0.5. For each simulated gene expression profile, we generated six biological replicates by randomly generating different error terms at each time point. Each array was standardized to have mean zero and unit standard deviation. We performed the simulation procedure a total of 100 times and summarized the results over these 100 simulations. Monte Carlo Markov Chain algorithm with Gibbs sampling was used to sample from the posterior distributions of parameters and draw inferences on the parameters for the simulated data. Each parameter was estimated as the mean of its posterior distribution based on an assumption for its prior distribution. Results were robust in most complex cases. In order to ensure that the sampling was from its equilibrium distribution, 2000 samples after 6000 burn ins were used for computation.

Various prior distributions, hyper-priors and initial values were tested for sensitivity analysis and to ensure the convergence of MCMC. The prior models were generated from normal distributions with means zero; the variances of the normal distributions were equivalent to that of the biological data. The hyper-prior distribution for means of the mixture model: *μ*_*i*_, *μ *~ *N*(0, *σ*^2^), *i *= 1,2,...,15, where *σ*^2 ^~ *InverseGamma*(1,0.001). Since we had no information on the precision (inverse of *σ*), small precisions were tested, such as 0.001, 0.01, 0.1 and 1 in order to the models to converge. For instance, when assigning uninformative distributions to these parameters, Inverse Gamma (0.001, 0.001), the models did not converge, but Inverse Gamma (0.1, 0.1) converged.

Sensitivity analysis was also conducted by varying the prior distributions. We consider several prior models with various hyper-priors and initial values. We first generated the prior model for the means (*μ*_*j*_), which followed conjugate normal priors *μ*_*j *_~ *Normal*(*γ*_*j*0_, $\sigma_{j0}^{2}$/*κ*_*j*0_), *j *= 1,...,*C*. The parameters of the prior on *μ*_*j *_were chosen from various values (e.g. 0.001, 0.01, 0.1) to give broad distributions, for instance, *γ*_*j*0 _~ *Normal*(0, 0.0001), $\sigma_{j0}^{2}$ ~ *Inverse Gamma *0.1, 0.1. Here, *γ*_j0 _is an initial guess on the mean in cluster j with *κ*_j0 _prior sample size reflecting strength of belief in the guess about the mean. The precision parameter in cluster j is given by *τ*_*j *_= 1/$\sigma_{j}^{2}$ ~ *Inverse Gamma (v*_*j*_*t*_*j*_/2, *v*_*j*_/2), where v_j _is the guess on the prior degrees of freedom, typically v_j _= 2 or lower. *t*_*j *_= 1/$s_{j}^{2}$ is the prior guess on the precision in component j, which will be tested from various values (e.g. 0.001, 0.01, 0.1). The best initial value will be chosen based on convergence of MCMC. For instance, when assigning uninformative distributions to these parameters, Inverse Gamma (0.001, 0.001), the models did not converge. It converged with Inverse Gamma (0.1, 0.1).

**Figure 5:**
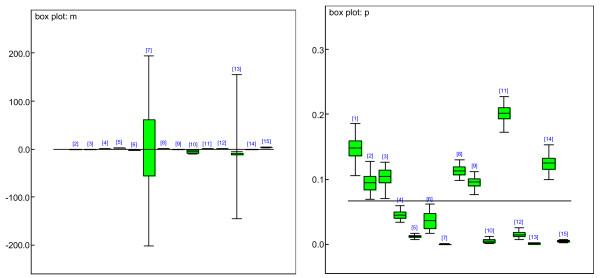
Simulation analysis: estimates of means for the mixture of normal distributions and estimates of proportions of the mixtures (15 clusters) based on simulated data. The left subfigure shows the estimated means for the mixtures of normal distributions. Almost all of them have very small variance, which indicate the appropriateness of the assumptions and estimates. The right subfigure shows the estimated proportions for each mixture group. Again, the variances are small.

Fig. 5 displays the simulation analysis for the estimates of means for the mixture of normal distributions and the estimates of proportions of the mixtures (15 clusters). The left subfigure sketches the estimated means for the mixtures of normal distributions. Almost all of them have very small variation, which indicate the appropriateness of the assumptions and estimates. Only two of the mixture groups have means with large variance. This may indicate groups that include outliers, which makes the estimation variance large (this may be eliminated by replacing Gaussian priors with student-t priors). However, these outliers are treated as a separate cluster in our model. Therefore, it does not affect the other clusters' estimates. The right subfigure sketches the estimated proportions for each mixture group. The variances are small. We see that most of the estimated gene expressions were around 0. Some of the genes were down-regulated with means less than 0; some of the genes were up-regulated with means greater than 0. This is biologically plausible.

**Figure 6:**
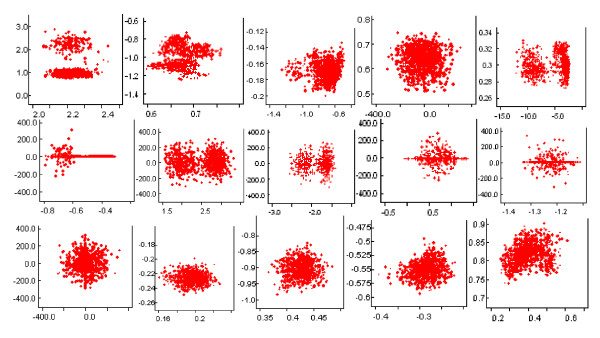
Simulation analysis: pair-wise scatterplots (for correlations) among the estimated means for clusters of the mixture model from simulated data. The mixture model assumes that there is no correlation among groups. These subfigures indicate that in our model, most of the means are not correlated to one another, which exactly obeys the assumptions; but some of them were weakly correlated. This may be due to outliers being treated as separate clusters. These clusters could have positive correlations that were not separable from other clusters. The Pearson correlation coefficients for pair-wise correlations (for clusters i vs. i+1, i = 1, 2,...,5) between two normal distributions were 0.0632, -0.0237, 0.1056, -0.0333, 0.0346, -0.0184, -0.0065, 0.0400, -0.0490, 0.0701, -0.0015, 0.3823, -0.4103, 0.3925, 0.3327, respectively.

To evaluate the model, which assumes that there is independence (no/weak correlation) among groups in the mixture model, we calculated Pearson correlation coefficients for pair-wise clusters given the above model. Fig. 6 shows the estimated correlations among the estimated means for the mixture of normal distributions of 15 clusters from the simulated data sets. These figures indicate that in our model, some of the clusters were independent (not correlated) of one another (for instance, Pearson correlation coefficients for cluster 1 versus 2; cluster 2 versus 3 and so forth were 0.0632, -0.0237, 0.1056, -0.0333, 0.0346, -0.0184, -0.0065, 0.0400, -0.0490, 0.0701, -0.0015, respectively), which exactly obeys the assumptions. However, there were a few of them that were correlated (cluster 11 versus 12, cluster 12 versus 13, 13 versus 14, 14 versus 15, the Pearson correlation coefficients were -0.3823, -0.4103, 0.3925, 0.3327, respectively). This may be due to the outliers being treated as separate clusters while these clusters could have positive correlations that were not separable from other clusters.

## Conclusion

In this paper, we present methodology for identifying genes that are differentially expressed over time and for identifying common profiles across different tissue types in order to address the inherent dependence between data observations when samples are collected in a time-ordered sequence, and also for increasing the power of the analysis. We have presented both Bayesian categorical model for estimating the proportion of the 'call', which are used for pre-screening genes and Hierarchical Bayesian Mixture model for identifying temporal differentially expressed gene expression and dynamic patterns. There are several advantages of the Hierarchical Bayesian Mixture model.

First, the model clusters gene expressions into up-regulation groups, down-regulation groups and no-regulation groups according to the mean expression level and the credible interval of posterior distributions of the gene expressions obtained for each mixture cluster. It provides probabilistic clustering in terms of the estimated posterior probabilities of component membership, which include the partitioning of the genes into C non-overlapping clusters, determined by the genes' highest estimated posterior probabilities of group membership. Second, it provides the uncertainty estimates of all the parameters through more accurate posterior intervals of differentially expressed genes versus less informative point estimates of p-values (hypothesis testing), which formed our base of making inferences about a sub-group of time related genes being differentially expressed (up or down regulated). Recall that providing 95% credible interval for gene based is equivalent to control the family wise error rate with significance level 0.05, which can be easily modified into controlling the false discovery rate to achieve less stringent results. Third, it provides us the posterior distribution of the clustered data as opposed to using standard normal distribution assumption models, that may not be valid or based on the empirical distribution from bootstrap or other resampling methods, which may have poor small sample properties.

Our model produces the clusters of non-differentially expressed genes and up/down regulated genes and also low-variation clusters and outliers. The model clusters the outliers and noise into one or several groups (clusters), which may make the preprocessing of excluding outliers before data analysis unnecessary in the future, although we have preprocessed our data in this paper for computational efficiency. Also, this model did not exclude the genes with expression profiles that had very low variation. These 'low-variation' genes may not provide any additional valid information about the time course of gene expression changes due to the drug effects, but they may be important to associate with certain pathways and their tiny fluctuations are exquisitely informative biological distinctions.

We have observed that our Bayesian estimation methods can deal with complex clustering situations and identify clusters of irregular shape, unequal size, or different dispersion. Furthermore, our developed model can deal with irregularly spaced time intervals and provides both the solutions for identification of differentially expressed time related genes and dynamic clustering. One important feature of our developed finite mixture model is that it is appropriate for further comparison and meta-analysis due to its ability to account for dependence among the genes and thus can summarize the concordance (intersection) among the tissues through the estimated posterior distributions as credible intervals. The resulting null standard deviations illustrate the precision of the resulting estimates.

Our hierarchical Bayesian model can be generalized and applied to any other time course microarray experimental data since it is based the dynamic patterns over all time points, similar to other function data analysis approaches [34-36]. One important difference between our approaches and other functional data analysis is that function analysis methods provide the estimates of the functions of the dynamic changes over time (most time nonlinear/cubic functions of the drug response), then cluster the similar genes with similar function based on the estimated functions; while Hierarchical Bayesian mixture model considers all the studied genes to be from mixture models and directly provides the estimates (including means standard deviation, credible intervals for each cluster), and the different dynamic clusters are automatically produced from the model.

Last, but more importantly, recall our model is hierarchical, by selecting different priors and hyper-priors, we also can achieve shrinkage and automatic selections effects, to further produce credible interval and posterior probabilities very close to 0 or 1. In this way no p-values; no type I error; no multiple comparisons are needed. This is one of the major advantages of our hierarchical mixture models. Some recent theoretical work has shown that there are similarities/equivalence between building Hierarchical Bayesian models with automatic shrinkage effects and designing optimization functions with L1- L2 norm or combined L1-L2 norm in statistical learning model in order to achieve automatic selection effects and avoiding the multiple testing/issues [10,37-40]. These studies have shown that they both are more efficient ways to deal with "curse of dimensionality" and turn the multiple testing problems for variable selection into an optimization problem in nonparametric setting, which are more computationally efficient and asymptotically optimal for high dimensional data.

## Authors' contributions

Both authors were involved in design, acquisition of data, analysis, interpretation of data, and manuscript preparation. Both authors have given final approval of the version to be published.

## Supplementary Material

Additional file 1 **WinBUGS code**. Use WinBUGS software to view WinBUGS code. Click here for file

## References

[B1] Egger M, Davey SG, Phillips AN (1997). Meta-analysis: principles and procedures. British Medical Journal.

[B2] Bailar JC (1997). The promise and problems of meta-analysis. New England Journal of Medicine.

[B3] DuMouchel WH, Harris JE (1983). Bayes methods for combining the results of cancer studies in humans and other species. Journal of the American Statistical Association.

[B4] Smith TC, Spiegelhalter DJ, Thomas A (1995). Bayesian approaches to random-effects meta-analysis: a comparative study. Stat Med.

[B5] Ghosh D, Barette T, Rhodes D (2003). Statistical issues and methods for meta-analysis of microarray data: A case study in prostate cancer. Functional Integrative Genomics.

[B6] Rhodes DR, Yu J, Shanker K, Deshpande N, Varambally R, Ghosh D, Barrette T, Pandey A, Chinnaiyan AM (2004). Large-scale meta-analysis of cancer microarray data identifies common transcriptional profiles of neoplastic transformation and progression. Proceedings of National Academy of Science.

[B7] Pan W, Wei W, Khodursky A (2008). A Parametric Joint Model of DNA-Protein Binding, Gene Expression and DNA Sequence Data to Detect Target Genes of a Transcription Factor. Pac Symp Biocomput.

[B8] Conlon EM, Song JJ, Liu A (2007). Bayesian meta-analysis models for microarray data: a comparative study. BMC Bioinformatics.

[B9] Liang Y, Kelemen A (2004). Hierarchical Bayesian Neural Network for Gene Expression Temporal Patterns. Stat Appl Genet Mol Biol.

[B10] Liang Y, Kelemen A (2005). Temporal Gene Expression Classification with Regularised Neural Network. International Journal of Bioinformatics Research and Applications.

[B11] Liang Y, Tayo B, Cai X, Kelemen A (2005). Differential and Trajectory Methods for Time Course Gene Expression Data. Bioinformatics.

[B12] Liang Y, Kelemen A (2006). Associating phenotypes with molecular events: a review of statistical advances and challenges underpinning microarray analyses. Journal of Functional and Integrative Genomics.

[B13] Liang Y, Kelemen A (2007). Bayesian State Space Model for Inferring and Predicting Transcription Profiles in Gene Expression. Biometrical Journals.

[B14] Efron B, Tibshirani R, Goss V, Chu G (2001). Empirical Bayes Analysis of a Microarray Experiment. Journal of American Statistical Association.

[B15] Pan W, Lin J (2003). A mixture Model approach to detecting differentially expressed genes with microarray data. Functional and Integrative Genomics.

[B16] Broet P, Lewin A, Richardson S, Dalmasso C, Magdelenat H (2004). A mixture model-based strategy for selecting sets of genes in multiclass response microarray experiments. Bioinformatics.

[B17] Kauermann G, Eilers P (2004). Modeling Microarray data using a threshold mixture model. Biometrics.

[B18] Liao J, Lian Y, Selvanayagam Z, Shih W (2004). A mixture Model approach for estimating the local false discovery rate in DNA microarray analysis. Bioinformatics.

[B19] Ghosh D (2004). Mixture models for assessing differential expression in complex tissues using microarray data. Bioinformatics.

[B20] Almon RR, Chen J, Snyder G, DuBois DC, Jusko WJ, Hoffman E (2003). In vivo Multi-Tissue Corticosteroid Microarray Time Series. Pharmacogenomics.

[B21] Jin JY, Almon RR, Dubois DC, Jusko WJ (2003). Modeling of corticosteroid pharmacogenomics in rat liver using gene microarrays. Journal of Pharmaceutical Experiment Therory.

[B22] Nimgaonkar A, Sanoudou D, Butte AJ, Haslett JN, Kunkel LM, Beggs AH, Kohane IS (2003). Reproducibility of gene expression across generations of Affymetrix microarrays. Bioinformatics.

[B23] Agresti A, Hitchcock DB (2005). Bayesian Inference for Categorical Data Analysis. Statistical Methods and Applications.

[B24] Congdon P (2002). Bayesian Statistical Modeling.

[B25] Fraley C, Raftery A (2002). Model-Based Clustering, Discriminant analysis, and Density estimation. Journal of American Statistical Association.

[B26] McLachlan GJ, Bean RW, Peel D (2002). A mixture model-based approach to the clustering of microarray expression data. Bioinformatics.

[B27] Medvedovic M, Sivaganesan S (2002). Bayesian infinite mixture model based clustering of gene expression profiles. Bioinformatics.

[B28] Teschendorff AE, Wang Y, Barbosa-Morais NL, Brenton JD, Caldas C (2005). A variational bayesian mixture modelling framework for cluster analysis of gene-expression data. Bioinformatics.

[B29] Lunn DJ, Thomas A, Best N, Spiegelhalter D (2000). WinBUGS – a Bayesian modelling framework: concepts, structure, and extensibility. Statistics and Computing.

[B30] Spiegelhalter D, Best N, Carlin B, Linde A (2002). Bayesian measures of model complexity and fit. Journal of Royal Statistical Society, B.

[B31] Do KA, Muller P, Tang F (2005). Bayesian mixture model for differential gene expression. Journal of the Royal Statistical Society, Series C.

[B32] Kim S, Tadesee MG, Vannucci M (2006). Variable selection in clustering via Dirichlet process mixture models. Biometrika.

[B33] Agresti A (2002). Categorical data analysis.

[B34] Ma P, Castillo-Davis CI, Zhong W, Liu JS (2006). A data-driven clustering method for time course gene expression data. Nucleic Acids Research.

[B35] Luan Y, Li H (2003). Clustering of time-course gene expression data using a mixed-effects model with B-spline. Bioinformatics.

[B36] Luan Y, Li H (2004). Model-based methods for identifying periodically regulated genes based on the time course microarray geneexpression data. Bioinformatics.

[B37] Tibshirani R (1996). Regression shrinkage and selection via the lasso. J Royal Statist Soc B.

[B38] Wang L, Zhu J, Zou H (2006). Doubly regularized support vector machine. Statistica Sinica.

[B39] Sun W, Cai T (2007). Oracle and adaptive compound decision rules for false discovery rate control. J American Statistical Association.

[B40] Liang Y, Kelemen A (2008). Statistical Advances and Challenges for Analyzing Correlated High Dimensional SNP Data in Genomic Study for Complex Diseases. Statistics Surveys.

